# Interaction between Heavy Water and Single-Strand DNA: A SERS Study

**DOI:** 10.3390/molecules27186023

**Published:** 2022-09-15

**Authors:** Chengshun Jiang, Yan Liu, Lianghua Wang, Feng Lu

**Affiliations:** 1 College of Pharmacy, Naval Medical University, Shanghai 200433, China; 2 College of Pharmacy, Fujian University of Traditional Chinese Medicine, Fuzhou 350122, China; 3 College of Basic Medical Sciences, Naval Medical University, Shanghai 200433, China; 4 Shanghai Key Laboratory for Pharmaceutical Metabolite Research, Naval Medical University, Shanghai 200433, China

**Keywords:** DNA structures, deuterium, hydrogen bonds, nucleobases, Raman spectroscopy

## Abstract

The structure and function of biological macromolecules change due to intermolecular deuterium bond formation or deuterium substitution with environmental D_2_O. In this study, surface-enhanced Raman spectroscopy (SERS) was used to detect interaction sites between D_2_O and ssDNA and their action mechanisms. SERS peaks of ssDNA changed with increasing D_2_O proportions, and the site of action mainly involved A and G bases, whose number strengthened the interaction between sequences and D_2_O and hence the SERS peak intensities. Fixing the number of A and G bases prevented changes in their positions from significantly altering the map. We also identified the interaction between ssDNA sequences that easily formed a G-quadruplex structure and D_2_O. The amplitude of the SERS peak intensity change reflected the ssDNA structural stability and number of active sites. These findings are highly significant for exploring genetic exchanges and mutations and could be used to determine the stability and structural changes of biological macromolecules.

## 1. Introduction

Heavy water (D_2_O) impacts the metabolism of organisms, and its consumption is hazardous to humans. Microbes and fish cannot survive in pure D_2_O or water containing a high proportion of D_2_O (>80%) and can die within hours of exposure. In contrast, light water with very little D_2_O, such as melted snow, stimulates biological growth. D_2_O is not believed to be toxic, but certain processes such as mitosis [1] require light water, and therefore, drinking only D_2_O can be detrimental to health. As D_2_O has an additional neutron, the mass of deuterium atoms in D_2_O is twice that of the hydrogen atoms in light water, which changes its properties. The various bonds formed by deuterium atoms are stronger than those of hydrogen atoms; for example, deuterium hydrogen bonds are 5% stronger than hydrogen bonds [2]. Furthermore, the transfer rate of the heavier deuterium atoms is much slower than that of hydrogen atoms, which slows down the biochemical reaction of deuterium atoms, interfering with the normal metabolism of organisms.

The metabolic process of DNA, the main genetic material of living organisms, is also affected by D_2_O. Presently, research on the effects of D_2_O on DNA, which has mainly focused on D_2_O as an isotope tracer, has revealed the metabolic kinetics of DNA by detecting the formed C-D bond [3,4]. Alternatively, adding an appropriate amount of D_2_O to the biological culture enables the elucidation of the effect of D_2_O on DNA metabolism by detecting changes in the metabolite levels or in the cell proliferation rate [5,6]. Drinking deuterium-depleted water has also been reported to reduce the breakage of single-stranded DNA (ssDNA) [7]. To date, studies have focused on examining the effect of D_2_O as a raw material for the synthesis of DNA and metabolic activities. D_2_O is used by organisms as a raw material in the synthesis of DNA, thereby affecting DNA replication and transcription processes. Furthermore, when DNA is placed in a D_2_O-containing environment, these molecules have a “weak interaction” that causes some groups to vibrate and chemical bond energy to change, which, in turn, affects the structure and function of DNA.

The effects of D_2_O on the stability of biomolecules are likely mediated by the following two mechanisms: one is the “solvent isotope effect”, where the hydrogen bond network in D_2_O is stronger than that in light water. The other mechanism is the “deuterium isotope effect”, which is caused by H/D isotope exchange between biomolecules and D_2_O [8]. D_2_O is 23% more viscous than H_2_O, which is important at the nanometer scale because the dynamic behavior of biomolecular machines is dominated by Brownian motion and solvent viscosity [9]. For double-stranded DNA (dsDNA), aromatic π-π stacking and hydrogen bonding between bases are the two key interactive forces that stabilize the double helix structure of DNA [10].

D_2_O affects hydrogen bonds; due to the rapid exchange of hydrogen and deuterium atoms, deuterium atoms are introduced into the double helix hydrogen bond structure of the DNA molecule [11]. This process affects the reading time of stored genetic information (the ratio of reading time of stored genetic information before and after hydrogen–deuterium exchange was 0.43), especially by changing the state of the genetic material (the “on” and “off” states of individual base pairs in DNA molecules) [11]. Replacing hydrogen atoms with deuterium atoms in the hydrogen bonds between the nitrogen base pairs of DNA molecules changes the frequency of the open states [12]. The frequency of open states is critical to molecular functionality, including promoting DNA–protein interactions between specific molecules during transcription, folding, and replication [12]. The probability of an open state between nitrogen bases in dsDNA depends on the concentration of deuterium in the liquid medium surrounding the molecule and the energy generated during hydrogen bond breakage [11]. The action of D_2_O on RNA is also mediated by the solvent isotope and deuterium isotope effects. The polyanionic nature of RNA induces a strong interaction with water, both H_2_O and D_2_O, destroying the stability of the folded configuration. However, it also prolongs the water bridge between residues, generating stronger water–water hydrogen bonds, which, in turn, penetrate the extended hydration shell of the RNA, producing a weaker RNA–water movement coupling, thereby providing stability [8]. After the hydrogen–deuterium exchange between RNA and D_2_O, the RNA stability is relatively increased because of the increase in the bond energy and overall mass of the chemical bond formed by the deuterium atoms.

Although exploring the interaction between D_2_O and ssDNA is essential, only a few studies have focused on their interactions [13,14]. Surface-enhanced Raman spectroscopy (SERS) is an ultra-sensitive molecular detection method that generates strong Raman scattering signals when the molecules are close to the rough surface of precious metals [15,16]. Numerous studies, such as those by Papadopoulou and Xu [17,18,19], have used SERS to detect and analyze DNA. Li [20] detected ssDNA sequences that could form G-quadruplex structures and further identified different G-quadruplex structures. Song [21] used Raman-Deuterium Isotope Probing (Raman-DIP) to identify antibiotic-resistant super bacteria in the Thames River. However, the study of D_2_O–ssDNA interactions based on SERS has not yet been reported.

The study of the interaction between D_2_O and ssDNA is of significance, as it can provide information on the effects of D_2_O on processes such as DNA replication and transcription; it also provides new insights into methods for the preservation, transportation, and stability research of oligonucleotide drugs (such as antisense oligonucleotides that contain ssDNA sequences). Therefore, in this study, SERS was used to explore the direct interaction between D_2_O and ssDNA. Furthermore, we identified and explored the sites and mechanism of action of both molecules and provided a relatively comprehensive description of the structural and functional changes of DNA exposed to D_2_O. To this end, the influence of D_2_O on the physiologically significant G-quadruplex structure was explored, which identified some potential mechanisms of the effect of D_2_O on ssDNA.

## 2. Results and Discussion

### 2.1. Detection of Random Sequences and Pure Base Sequences

**Figure 1:**
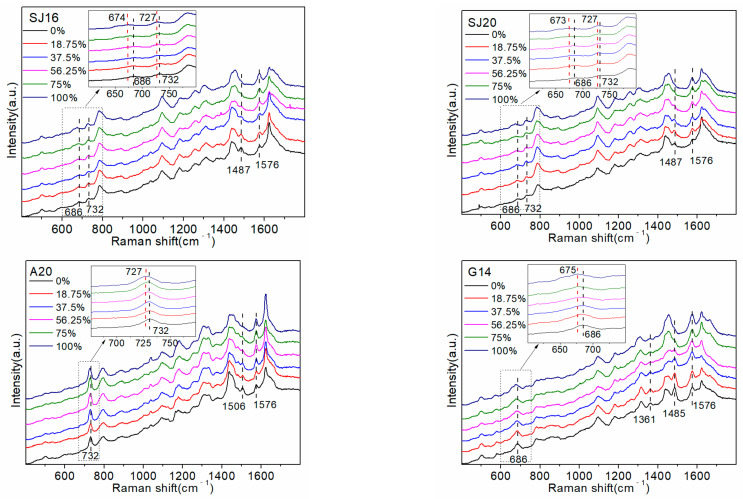

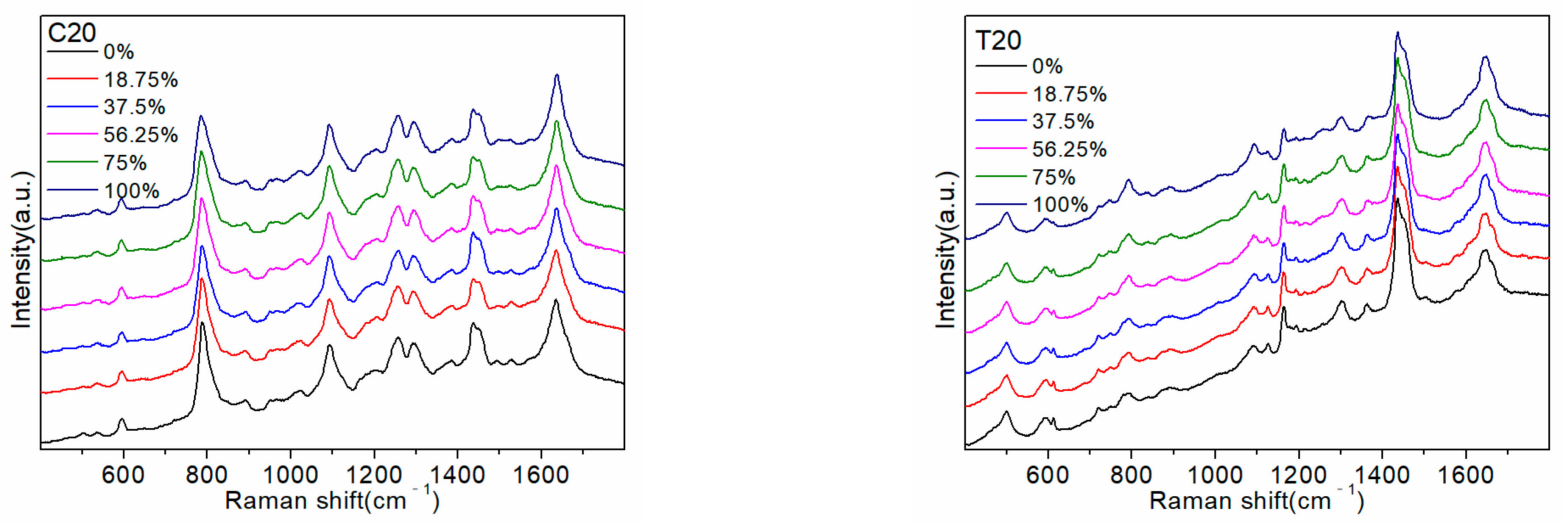
Surface-enhanced Raman spectroscopy (SERS) spectra of random and pure base sequences.

To explore the interaction between D_2_O and ssDNA, two random sequences, SJ16 and SJ20, were detected first. The specific sequences SJ16 and SJ20 represent are shown in Table 1. Subsequently, pure base sequences A20, G14 (the number of consecutive G bases did not exceed 14), C20, and T20 were detected to attribute the SERS peaks and determine the common active sites between them. The results are shown in Figure 1.

Replacing the atoms of the molecular vibrating group with their isotopes shifted the vibration frequency and changed the spectral intensity. Figure 1 shows the 686 cm^−1^ signal belonging to the G base ring respiratory vibration peak [19,22,23], which gradually shifted to 675 cm^−1^ as the D_2_O proportion increased. This likely occurred because some hydrogen atoms in the G base were replaced by deuterium atoms, which increased the overall mass, reduced the vibration frequency, and caused a red shift in the peak. The peak at 732 cm^−1^ was attributed to the A base ring respiratory vibration peak [19,23,24], which gradually decreased with the increase in the proportion of D_2_O, and the frequency shifted to 727 cm^−1^ (100% D_2_O).

The decrease in the intensity of the peak at 732 cm^−1^ occurred because D_2_O has a higher viscosity than ordinary water, making it more conducive to nucleic acid folding [8]. Therefore, the A base is squeezed by the surrounding groups, its ring breathing vibration is affected, and the peak intensity is weakened. The frequency shift is affected by the H/D isotope exchange, which increases the overall mass and decreases the vibration frequency. Therefore, there was a slight red shift in the peak position. In the pure A base, the peak at 732 cm^−1^ did not weaken because all sequences were A bases, which would be difficult to fold. The respiratory vibration of the A base ring was only slightly affected by the environmental D_2_O, and therefore, the peak at 732 cm^−1^ did not change markedly.

The peak at 1485 cm^−1^ was attributed to the hydrogen bond formed by N7 on the G base and the surrounding H_2_O/D_2_O [20,22]. Gradually decreasing the surrounding H_2_O concentration (i.e., gradually increasing the D_2_O concentration) increases the number of deuterium–hydrogen bonds formed by N7 and D_2_O. This action causes the isotopic effect to weaken the vibration tendency of the deuterium–hydrogen bonds and the peak intensity. As the hydrogen bonds formed between both molecules are not tetrahedral, there is no peak frequency shift [25]. The peak intensity at 1576 cm^−1^ increased with an increasing proportion of D_2_O in both the A20 and G14 sequences, but the change in the G14 sequence was more obvious.

Therefore, the change in the peak at 1576 cm^−1^ was caused by the interaction between the NH_2_ group on the A and G bases in the sequence and D_2_O. Furthermore, deuterium atoms do not directly participate in the formation of hydrogen bonds, which are still NH…O, and the O-D bond in D_2_O has higher energy and a shorter bond length than the O-H bond in H_2_O. Therefore, the NH…O bond polarizability, Raman activity, and Raman strength increase.

The following two interesting phenomena were observed: the peaks at 1506 cm^−1^ and 1361 cm^−1^ changed with increasing D_2_O proportion in pure base sequences A20 and G14. However, these changes were not evident in the SERS spectra of random sequences, i.e., SJ16 and SJ20. The 1506 cm^−1^ peak in the SERS spectrum of the A20 sequence was attributed to the A base. As the number of A bases in the sequences SJ16 and SJ20 (three and four, respectively) and the Raman intensity of this peak was relatively low, it did not appear in the SERS spectra of SJ16 and SJ20. Increasing the number of A bases in the sequence, such as with the sequence A12GTC in 3.3 (including 12 A bases), caused the peak at 1506 cm^−1^ to appear in the SERS spectra, and the intensity increased with the increasing proportion of D_2_O in the environment. A comparison of the SERS spectra of SJ16, SJ20, and the four pure base sequences showed that the 1361 cm^−1^ peak was attributable to the G and T bases [24]. Increasing the proportion of D_2_O did not change the peak of the pure T base at 1361 cm^−1^, whereas the peak intensity of the pure G base changed significantly, but the change was very weak in the SJ16 and SJ20 sequences. This likely occurred because the number of G bases in the sequence was relatively low (seven and eight, respectively). Although a total of eight G bases is small, it accounts for 40% of all bases, and the intensity of the peak in G14 was not high. Therefore, the change caused by eight G bases would not be very obvious. In the G12ATC sequence of the follow-up experiment, there were 12 G bases, accounting for 60% of the total number of bases, and the peak intensity change of 1361 cm^−1^ was more visible than that of SJ16 and SJ20. However, the apparent degree was still less than that of G14 (100% G base) at the 1361 cm^−1^ peak.

**Figure 2:**
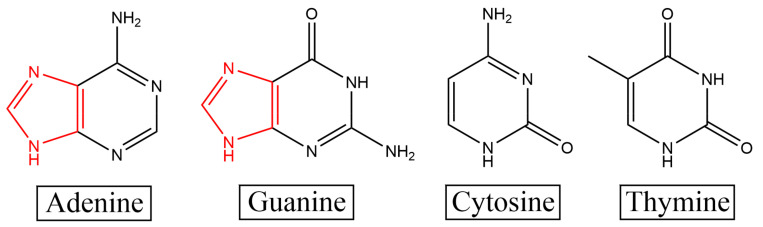
Structural diagram of the four bases. Among them, A bases and G bases contain five-membered ring structures, which increase the sites of interaction with D_2_O.

In summary, the Raman peaks that changed with an increasing proportion of D_2_O in the random sequence SERS spectrum were almost all related to the A and G bases. Therefore, we preliminarily inferred that the interaction site of D_2_O and DNA was mainly at the A and G bases. A comparison of the structures of the four bases determined that the A and G bases had a unique five-membered ring structure (as shown in Figure 2), which increased the site of interaction with water. Owing to their hydrophobicity, the bases in DNA gather in water to reduce contact with water [26]. As the number of sites on the A and G bases that interact with water is higher than that on the C and T bases, the A and G bases are less hydrophobic (more hydrophilic) than the C and T bases, and their ability to bind to H_2_O is stronger. C and T bases have fewer interaction sites with H_2_O than A and G bases; therefore, they are more hydrophobic, which is not conducive to interaction with H_2_O. When the concentration of D_2_O in the environment increases, more sites of action not only increase the number of hydrogen–deuterium exchanges between A and G bases and D_2_O and the number of deuterium–hydrogen bonds formed, but also reduce the total energy of the formed complex and thus improve its stability. Simultaneously, they increase the influence of D_2_O on the group vibration in the A and G bases, which is conducive to the capture of the SERS instrument. As the C and T bases and D_2_O interact with fewer sites, it not only reduces the number of hydrogen–deuterium exchanges and the formation of deuterium–hydrogen bonds between the two but also makes the total energy of the complex higher and lowers its stability. This leads to a reduction in the influence of D_2_O on the group vibration of C and T bases, which is not conducive to the capturing of changes by the SERS instrument. Therefore, the SERS peaks related to the C and T bases barely changed, except the 1170 cm^−1^ peak belonging to T bases was weakened.

### 2.2. Detection of Only C and T Base Sequences

**Figure 3:**
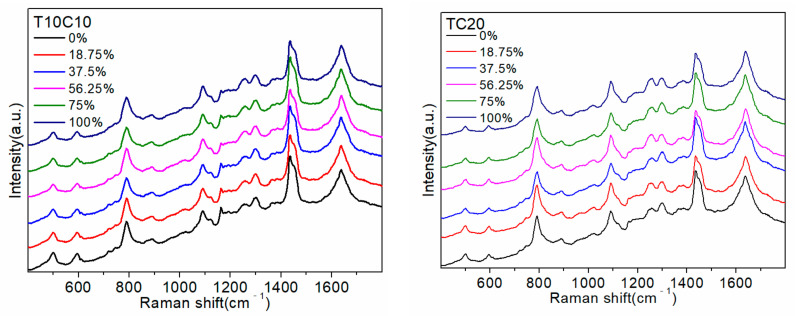
Detection of T10C10 and TC20 sequences.

To verify that the site of action between D_2_O and DNA identified in Section 2.1 involves mainly A and G bases, two sequences, T10C10 and TC20, containing only C and T bases were designed. The two sequences were subsequently detected using the same process, and the results are shown in Figure 3.

Figure 3 shows that the T10C10 and TC20 sequences only contained C and T bases, with the same number of each, but their different order resulted in different SERS spectra. However, the SERS spectrum was almost unchanged—only the intensity of the 1170 cm^−1^ peak showed a small change with an increase in the proportion of D_2_O in the environment where the sequence was located. Consequently, the SERS spectrum containing only C and T base sequences was rarely affected by D_2_O, which also verified the conclusion reached in Section 2.1 that the site of interaction between D_2_O and DNA was mainly at the A and G bases.

### 2.3. Effect of Number and Position of G Base in Sequence

**Figure 4:**
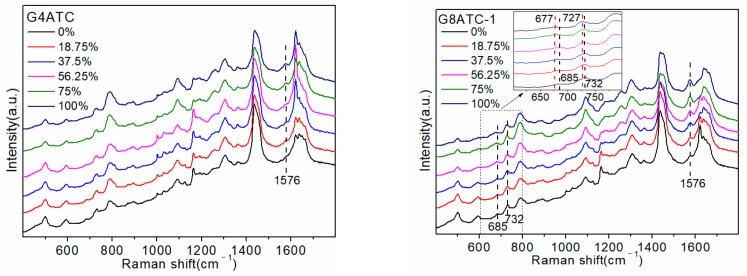

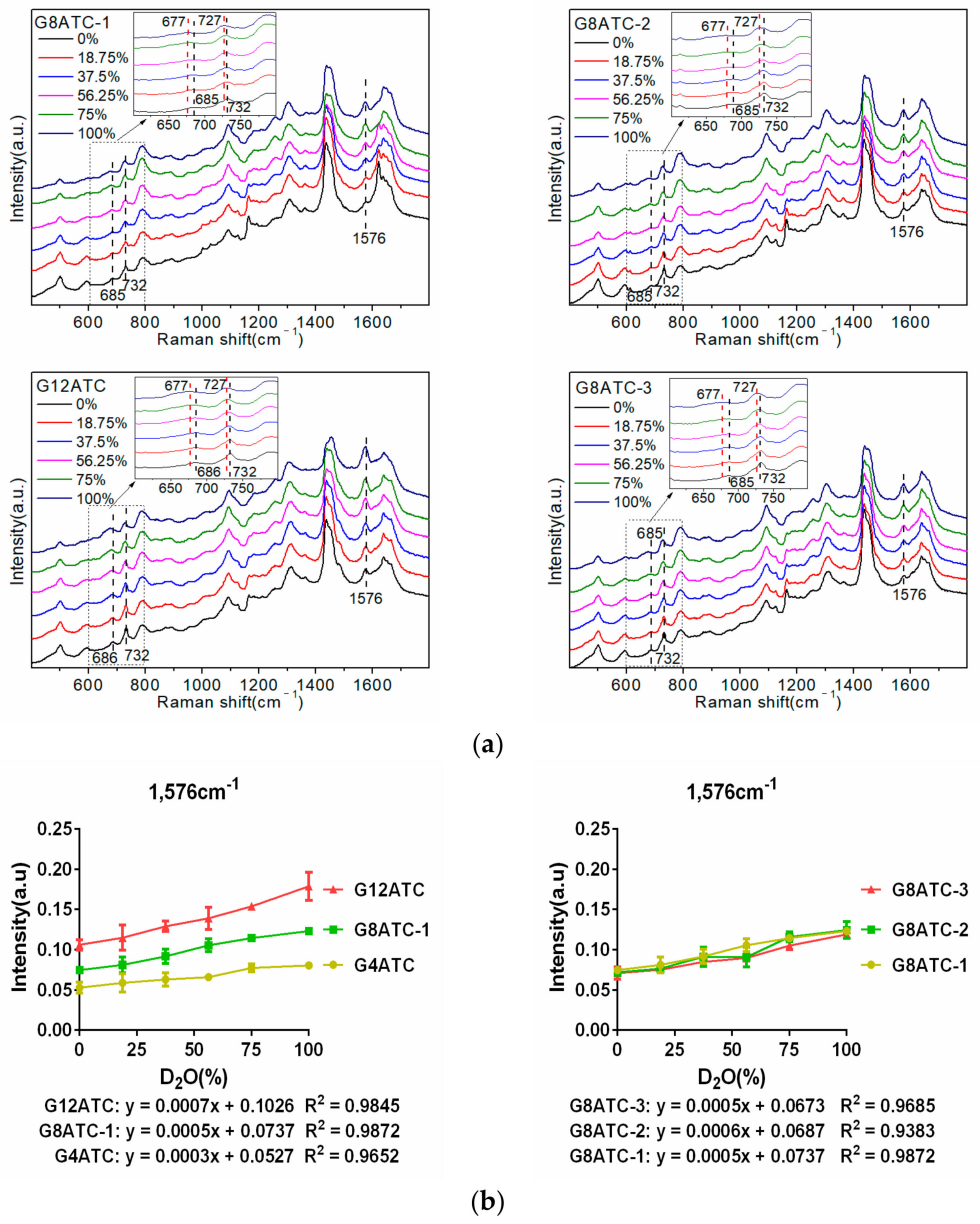
SERS detection of sequences. (**a**) G4ATC, G8ATC (-1, -2, -3), and G12ATC sequences detected at different concentrations of heavy water (D_2_O). (**b**) Trend of changes in intensity of the peak at 1576 cm^−1^ in G4ATC, G8ATC-1, G12ATC, G8ATC-1, G8ATC-2, and G8ATC-3 sequences with different concentrations of D_2_O and linear equation of the peak intensity at 1576 cm^−1^ related to heavy water (D_2_O) proportion.

To explore the effects of the number and position of G bases in the sequence, the following five sequences were designed: G4ATC, G8ATC-1, G8ATC-2, G8ATC-3, and G12ATC, as shown in Table 1. All sequences contained 20 bases, and the number of G bases was 4, 8, 8, 8, and 12, respectively. The four G and eight G bases replaced the C bases that did not interact with D_2_O. The three G8ATC (-1, -2, and -3) sequences contained the same number and types of bases, but the order of arrangement was different. The five ssDNAs detected are shown in Figure 4a.

The increase in the proportion of D_2_O produced a Raman peak in the spectrum that was consistent with that of SJ16 and SJ20. Specifically, the 686 cm^−1^ and 732 cm^−1^ peaks had a red shift, and the intensities of the 732 cm^−1^ and 1485 cm^−1^ peaks gradually weakened, whereas that of the peak at 1576 cm^−1^ gradually increased. The largest change was in the intensity of the 1576 cm^−1^ peak. Using the peak intensity as a reference, we explored the influence of the number and position of G bases in the sequence on their interaction with D_2_O. The change in the peak intensity with the increasing proportion of D_2_O in the different sequences and the linear relationship between the peak intensity and the proportion of D_2_O are shown in Figure 4b.

The G4ATC, G8ATC-1, and G12ATC sequences are all 20 bases long. The extra G bases of the latter two were replaced by C bases that did not interact with D_2_O; therefore, the changes in the three maps were mostly caused by the increase in the number of G bases. In the SERS spectrum, when the proportion of D_2_O was 0%, the difference in the SERS spectra of the three sequences was mainly reflected in the peaks at 1485 cm^−1^ and 1576 cm^−1^, which were both attributed to the G base. In contrast, the peak at 732 cm^−1^ attributed to the A base showed almost no difference. Figure 4b shows that the 1576 cm^−1^ peak intensity of the three sequences was positively correlated with the number of G bases, and the slopes of the linear equations of the three sequences were 0.0003, 0.0005, and 0.0007, respectively, which showed an upward trend. Specifically, increasing the number of G bases also increased the amplitude of the intensity of the 1576 cm^−1^ peak with an increase in the proportion of D_2_O. Therefore, the greater the number of G bases, the stronger the interaction between the sequence and D_2_O.

Differences in the arrangement of sequences produced slight variations in the intensities of some peaks in the SERS spectra of the three G8ATC sequences. For example, for the 732 cm^−1^ peak, a D_2_O proportion of 0% produced an A8GTC-2 sequence that had a stronger intensity than the A8GTC-1 sequence. However, under D_2_O conditions, the trend of peak intensity and displacement in the three sequences was the same as the proportion of the D_2_O increase. Figure 4b shows that the 1576 cm^−1^ peak intensity of the three G8ATC sequences was between 0.075 and 0.135, and the slopes of the linear equation were almost equal (0.0005–0.0006). Therefore, when the number of G bases in the sequence was constant, the difference in the amplitude of the 1576 cm^−1^ peak intensity of the three sequences was very small and was smaller than the difference caused by the difference in the number of G bases. We believe that this phenomenon occurred because the volume of the D_2_O molecules was small relative to the base and ssDNA sequences in the system. Regardless of the position of the base in the sequence, the active site of both molecules was always “exposed”, and the small D_2_O molecule always localized to and interacted with the active site. Therefore, the sequence interaction with D_2_O was not affected by the positions of the A and G bases in the sequence.

### 2.4. Effect of Number and Position of A Base in Sequence

**Figure 5:**
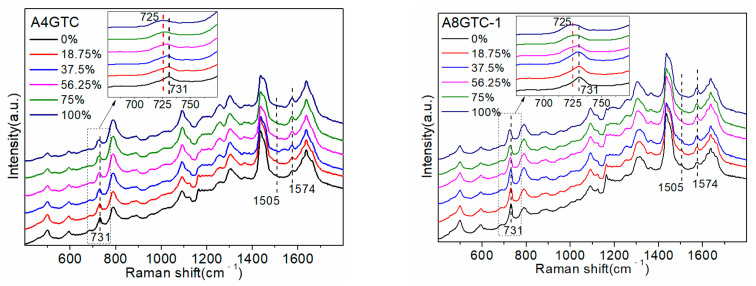

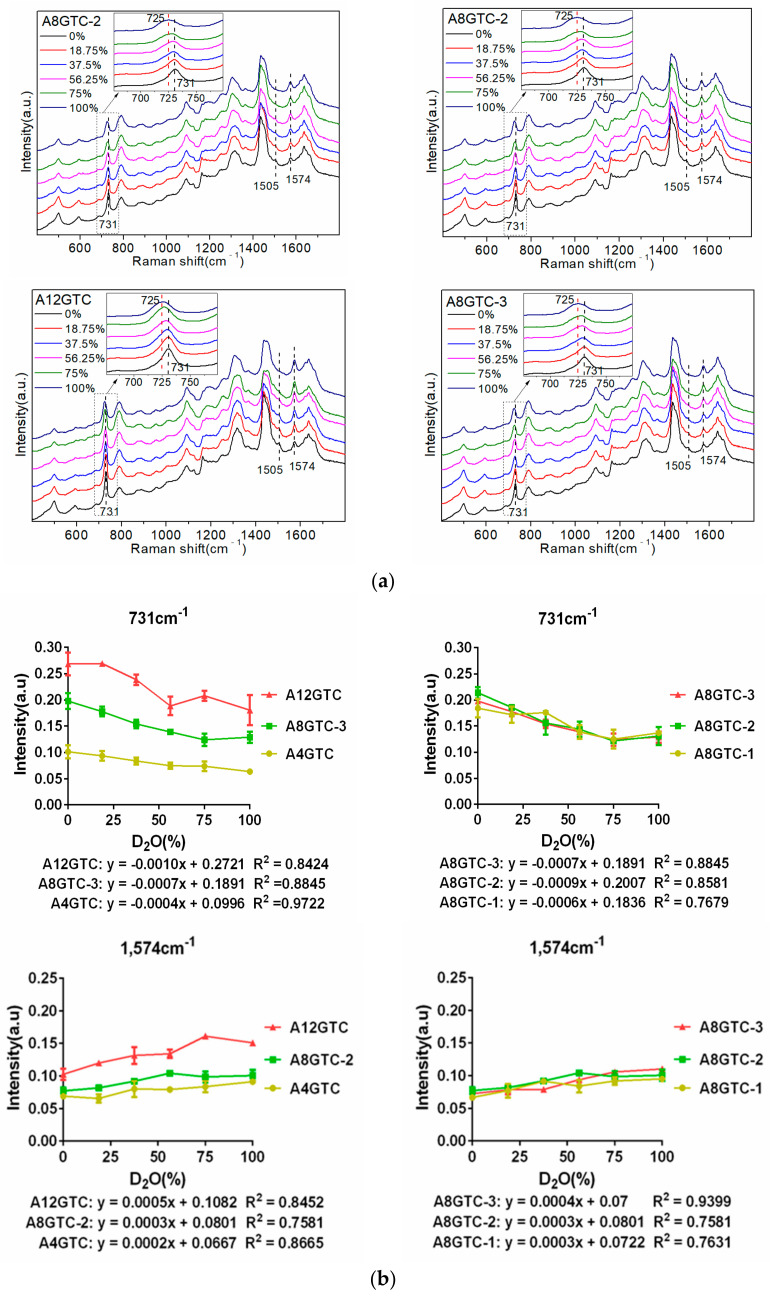
SERS detection of sequences. (**a**) A4GTC, A8GTC (-1, -2, and -3), and A12GTC sequences in heavy water (D_2_O) with different concentrations. (**b**) Trend of changes in intensities of the peaks at 731 and 1574 cm^−1^ of A4GTC, A8GTC-2(-3), A12GTC, A8GTC-1, A8GTC-2, and A8GTC-3 sequences in different concentrations of D_2_O and linear equation of peak intensities at 731 and 1574 cm^−1^ with respect to heavy water (D_2_O) proportion.

In addition, to explore the influence of the number and position of the A bases, the following five sequences were designed: A4GTC, A8GTC (-1, -2, and -3), and A12GTC; the specific sequences they represent are shown in Table 1. All sequences contained 20 bases, and the number of A bases was 4, 8, 8, 8, and 12, respectively. The increase in four and eight A bases replaced the C bases that did not interact with D_2_O. Three A8GTC (-1, -2, and -3) sequences contained the same number and types of bases, but they were arranged in a different order. The five ssDNAs detected are shown in Figure 5a.

As all sequences had the same number of G bases, their position did not affect the intensity of the 1574 cm^−1^ peak, which was only altered by a change in the A base. Increasing the proportion of D_2_O resulted in changes in the Raman peak spectrum that were consistent with those observed in the SJ16 and SJ20 spectra. Specifically, the peak at 731 cm^−1^ had a red shift, and the intensities of the 731 cm^−1^ and 1505 cm^−1^ peaks gradually decreased, whereas that of the 1574 cm^−1^ peak gradually increased. We used the peaks at 731 cm^−1^ and 1574 cm^−1^, which had the largest intensity changes, as references to explore the influence of the number of A bases and their positions in the sequence on the interaction between the sequence and D_2_O. The trend of change in the peak intensities at 731 cm^−1^ and 1574 cm^−1^ with increasing D_2_O proportion and the linear equation of the peak intensity with respect to the proportion of D_2_O are shown in Figure 5b.

The A4GTC, A8GTC-2, and A12GTC sequences were all 20 bases long. The extra A bases in the A8GTC-2 and A12GTC sequences were included by replacing the C bases that do not interact with D_2_O. Therefore, the changes in the three SERS maps could almost all be attributed to an increase in the number of A bases. Figure 5b shows that the 731 cm^−1^ peak intensity of the three sequences was positively correlated with the number of A bases and decreased with an increase in the D_2_O proportion. The linear equation slopes of the peak intensity with respect to the proportion of D_2_O were −0.0004, −0.0007, and −0.0010, respectively, and the absolute value showed an increasing trend. Moreover, as the number of A bases increased, the reduction in the intensity of the 731 cm^−1^ peak increased with an increase in the proportion of D_2_O. The 1574 cm^−1^ peak intensity of the three sequences was positively correlated with the number of A bases and increased with an increase in the proportion of D_2_O. The linear equation slopes of the peak intensity with respect to the proportion of D_2_O were 0.0002, 0.0004, and 0.0005, respectively: i.e., they showed an upward trend. Specifically, as the number of A bases increased, the magnitude of enhancement of the intensity of the 1574 cm^−1^ peak increased with an increase in the proportion of D_2_O. Therefore, the greater the number of A bases in the sequence, the stronger the interaction between the sequence and D_2_O. In Section 2.3, it was shown that the linear equation slopes of the 1576 cm^−1^ peak intensity of the G4ATC, G8ATC, and G12ATC sequences increased with the proportion of D_2_O, i.e., 0.0003, 0.0005, and 0.0007, respectively. Importantly, these values were greater than the linear equation slopes of the 1574 cm^−1^ peak intensity of the A4GTC, A8GTC-2, and A12GTC sequences and the proportion of D_2_O. Therefore, the interaction between the G base and D_2_O was stronger, causing a greater change in the intensity of the 1576 cm^−1^ peak than that caused by the A base.

Differences in the order of arrangement induced slight differences in the intensities of some peaks of the SERS spectra of the three A8GTC sequences. However, the peaks and trends of changes for the three sequences with respect to the proportion of D_2_O were consistent. Furthermore, the 731 cm^−1^ and 1574 cm^−1^ peaks with pronounced changes were similarly analyzed. The intensity of the 731 cm^−1^ peak of the three sequences was between 0.11 and 0.21 (linear equation slopes: −0.0006, −0.0009, and −0.0007), and that of the 1574 cm^−1^ peak was between 0.06 and 0.11 (linear equation slopes: 0.0003, 0.0003, and 0.0004). The absolute slopes of the linear equation were not significantly different, and the slopes of the 1574 cm^−1^ peak were almost equal. Therefore, when the number of A bases in the sequence was constant, the difference in the peak intensities of the three sequences at 731 cm^−1^ and 1574 cm^−1^ was very small (smaller than the difference induced in response to variations in the number of A bases).

Investigation of the number and position of the A and G bases in the sequence suggests that the interaction between D_2_O and sequences with the same length was affected by the change in the number of A and G bases. In contrast, changes in the position of the A and G bases had less pronounced effects. Owing to the volatility of SERS, for sequences that cannot be folded into specific secondary structures, such as G-quadruplexes and i-motifs, their interaction with D_2_O is unaffected by the position of A and G bases.

### 2.5. Interaction between G-Quadruplex Sequence and D_2_O

We concluded that D_2_O mainly interacts with the A and G bases in the sequence and is mainly affected by their number rather than location, provided that the sequence does not form specific secondary structures. Therefore, we investigated the outcome when the sequence formed a secondary structure.

We selected the G-quadruplex as the paradigm for investigating the interaction between D_2_O and the ssDNA secondary structure. G-quadruplexes are unique secondary structures formed by guanine-rich nucleic acids. Considerable in vitro biophysical and structural evidence supports the formation of a G-quadruplex [27,28]. Computer research and sequencing methods have revealed the ubiquity of G4 sequences in the gene regulatory regions of different genomes, including human genomes [29,30]. Experiments using chemical, molecular, and cellular biological methods have demonstrated the presence of G4 sequences in chromatin, DNA, and RNA, and the formation of G-quadruplexes is very important for key biological processes, such as transcription, translation, genome instability, and cancer development [31,32,33].

**Figure 6:**
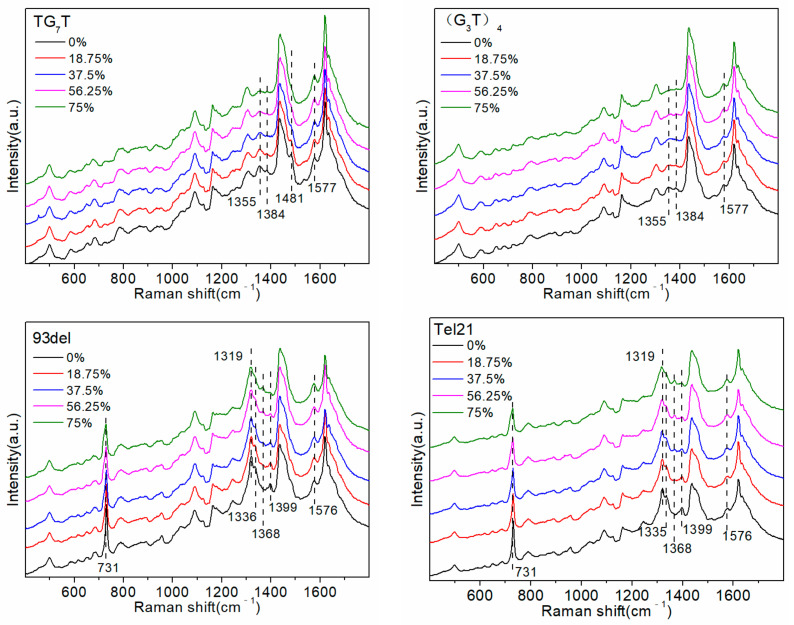

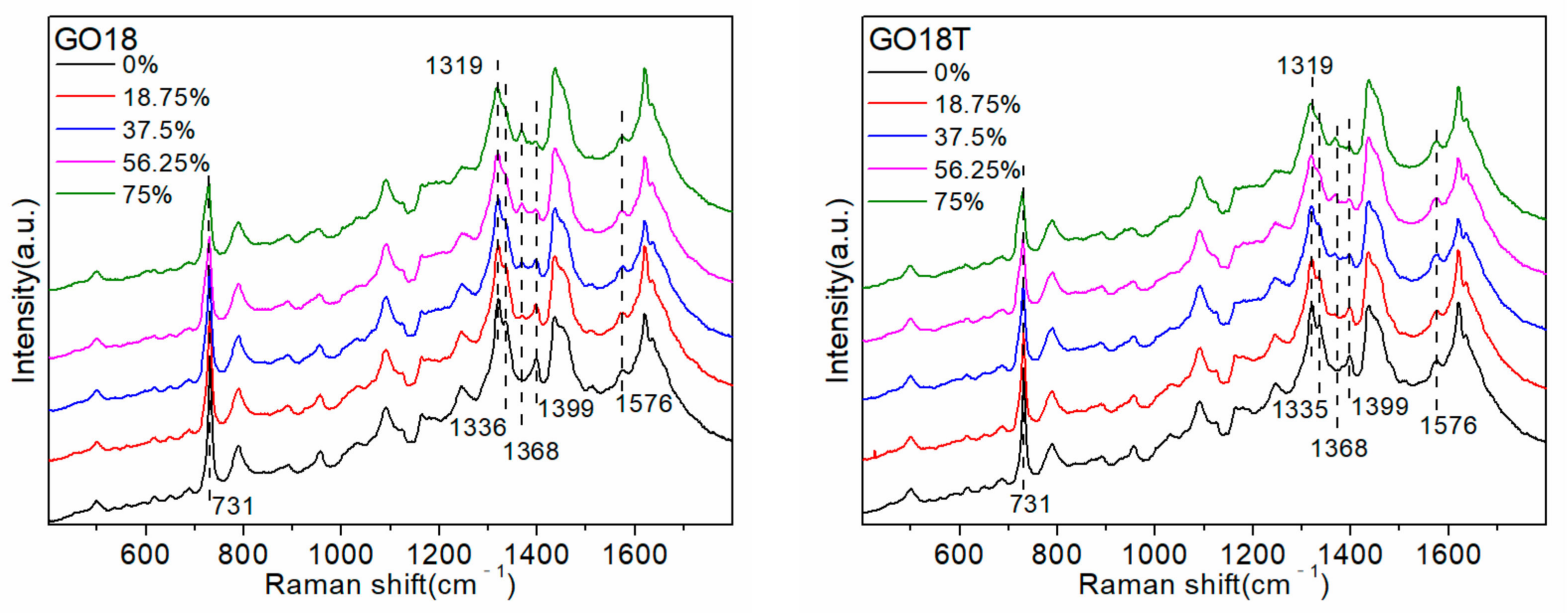
Detection of the G-quadruplex sequence in the presence of different concentrations of heavy water (D_2_O).

In addition, G-quadruplexes can also serve as potential therapeutic targets for human diseases, thereby improving the therapeutic effect on diseases such as cancer and genetic diseases [34]. Therefore, it is necessary to explore the interaction between D_2_O and G4 sequences, which would contribute to enhancing our understanding of the structure and properties of G-quadruplexes. Furthermore, such studies would provide partial theoretical guidance for further studies of various biological processes that require the simultaneous involvement of D_2_O and G-quadruplexes. We synthesized six sequences that form G-quadruplexes, i.e., (G_3_T)_4_, TG_7_T, Tel21, 93del [20], GO18, and GO18T [35] (Table 1 shows the specific sequences they represent) and explored their interaction with D_2_O. The results are shown in Figure 6.

**Figure 7:**
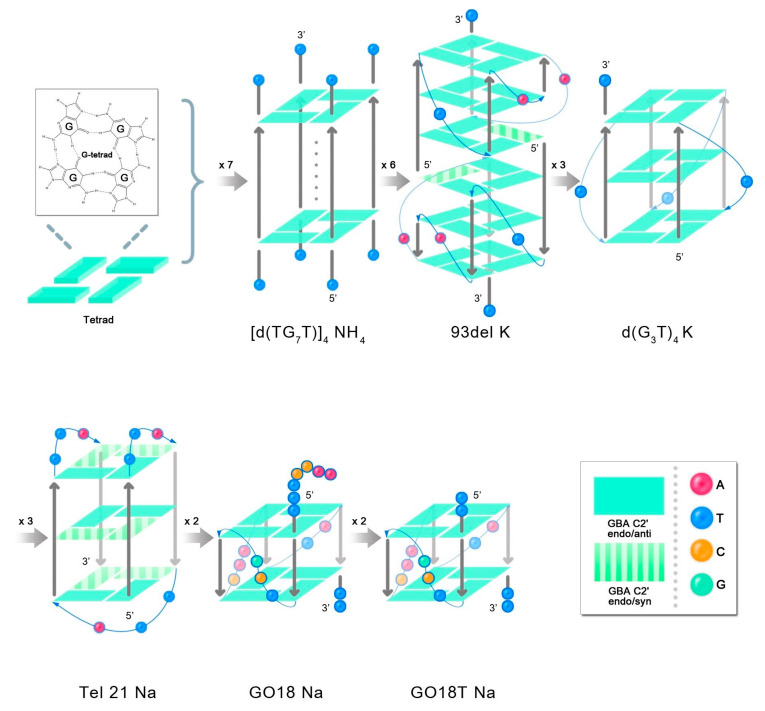
G-quadruplex structures formed by TG_7_T, 93del, (G_3_T)_4_, Tel21, GO18, and GO18T. In the presence of the corresponding cation, sequence d(TG_7_T) forms a tetramolecular structure, all chains are arranged in the 5′-3′ direction, and all GBAs adopt the trans conformation. 93del K: Parallel embedded bimolecular interlocking structure with cis–trans alternating GBAs and multiple loop structures; d(G_3_T)_4_ K: parallel single-molecule structure with three loop structures, and all GBAs adopt the trans conformation; Tel21 Na: antiparallel monomolecular structure, GBAs with cis–trans alternate conformation, three loop structures; GO18 Na and GO18T Na: parallel single-molecule structure with three loop structures, and all GBAs adopt the trans conformation.

After adding a cationic acetate buffer (pH 4.5), the ssDNA formed a G-quadruplex structure [20,23], which is shown in Figure 7, thereby inducing partial changes in the sites where it interacted with D_2_O. First, the intensity of the 1576 cm^−1^ peak changed very slightly with a change in the D_2_O proportion, as the sites that originally interacted with D_2_O were involved in the formation of intramolecular hydrogen bonds, forming a G-quadruplex structure, and showed no intermolecular hydrogen bonding interactions with D_2_O. Although the A base also interacts with D_2_O to induce an alteration in the intensity of the 1574 cm^−1^ peak, the number of A bases in the G4 sequence is often very small. The smaller the number of A bases, the weaker the interaction with D_2_O. Therefore, there was almost no change in the intensity of the 1576 cm^−1^ peak with a change in the proportion of D_2_O. The 1399 cm^−1^ peak, attributable to the T base [20,23], only appeared in the G-quadruplex sequence, and its intensity decreased with the increasing proportion of D_2_O. This was because after the G-quadruplex structure was formed, T bases were located outside the G-tetrad or even in the G-quadruplex, which enabled them to move closer to the surface of the silver nanoparticles. Furthermore, the SERS signal of the T bases was enhanced more strongly, and therefore, the signal was revealed in the SERS spectra. Moreover, compared to that of the sequence that did not form the G-quadruplex structure, the intensity of the 731 cm^−1^ peak of the G-quadruplex sequence was exceptionally strong ([G_3_T]_4_ and TG_7_T did not contain A bases, and this shift was not associated with any peak). This observation was also likely due to the fact that after the G-quadruplex structure is formed, more A bases are located outside the G-tetrad or even the G-quadruplex. Consequently, they are located closer to the surface of the silver nanoparticles, and the A base SERS signal is more strongly enhanced. Therefore, in the G-quadruplex sequence containing A and T bases, the intensities of the 1399 cm^−1^ and 731 cm^−1^ peaks reflected the number and stability of G-quadruplex structures. The greater the number and stability of G-quadruplexes, the greater the strength of these two peaks. Figure 6 shows that as the proportion of D_2_O increased, the intensities of the 1399 cm^−1^ and 730 cm^−1^ peaks were gradually reduced, indicating that D_2_O reduced the number and stability of G-quadruplex structures.

In addition, increasing the proportion of D_2_O in the solution gradually resulted in reduced intensities of the 1319 cm^−1^ and 1336 cm^−1^ peaks, whereas that of the 1368 cm^−1^ peak gradually increased. The positions and intensities of the Raman bands in the ranges 550–700 cm^−1^ and 1300–1380 cm^−1^ have been reported to be related to the glycosidic bond angle (GBA) conformation of all G, A, and T bases [36]. The number and type of GBAs contained in different G-quadruplexes are different, resulting in differences in the Raman shifts attributed to the GBA structure in the Raman signal of the G4 sequence. In the literature, the 1319 cm^−1^ and 1336 cm^−1^ peaks are assigned to dG C2′-endo/anti, and the 1368 cm^−1^ peak is assigned to dG C2′-endo/syn; (G_3_T)_4_ and TG_7_T does not contain the C2′-endo/syn structure, and therefore, the 1355 cm^−1^ peak was assigned to the C2′-endo/anti-structure [20,22]. The changes in the peak intensities at 1319 cm^−1^, 1336 cm^−1^, 1355 cm^−1^, and 1368 cm^−1^ indicated alterations in the C2′-endo/anti and C2′-endo/syn structures of the G base, which further reflected changes in the syn/anti-GBA conformation of the G-quadruplex.

In the G-quadruplex structure formed by (G_3_T)_4_ and TG_7_T, all sequences were arranged 5′-3′, and all GBAs adopted a trans conformation. Increasing the proportion of D_2_O gradually resulted in the reduced intensity of the 1355 cm^−1^ peak, indicating that the number of GBAs in the trans conformation gradually decreased, and the number or stability of G-quadruplexes decreased. In the G-quadruplex structure formed by the sequences of 93del, Tel21, GO18, and GO18T, the arrangement direction of the DNA strands was not uniform, and a few cis conformations were formed in the GBAs. (In the G-quadruplex structure formed by GO18 and GO18T, GBA is a trans conformation, but the cis-GBA signal also appears in the spectrum. Our guess is that their two-layer G-quadruplex structure is very close due to π-π stacking, which squeezes the three bases on the connecting chain, presenting a conformation quite similar to cis-GBA.) The SERS signal of the trans conformation GBAs was dominant in the SERS spectrum of the four G-quadruplex sequences. As the proportion of D_2_O increased, the intensities of the 1319 cm^−1^ and 1336 cm^−1^ peaks greatly decreased, indicating that the number of GBAs in the trans conformation decreased, whereas the intensity of the 1368 cm^−1^ peak, representing the cis conformation of GBAs, only slightly increased, indicating that the quantity or stability of the G-quadruplexes decreased.

In summary, D_2_O was not conducive to the formation and stability of the G-quadruplex structure, which is inconsistent with the conclusion that D_2_O stabilizes the folded structure of nucleic acids, as reported in the literature [8]. We believe that because of the increased viscosity, D_2_O induces DNA to fold, but this fold is not necessarily fixed and only randomly forms a part of the intramolecular hydrogen bonds. In the presence of the cationic buffer, the G-quadruplex structure formed by a specific sequence was fixed. However, D_2_O negatively affected this “fixation”, as it increased the tendency of DNA to fold into other random structures.

Therefore, for ssDNA that cannot form a specific structure, D_2_O promotes its random folding, whereas for ssDNA that does form a specific structure, D_2_O inhibits the process of folding into specific structures. This discovery has important implications for certain biological processes involving G-quadruplexes. For example, the human chromosome telomere sequence contains numerous G-quadruplex structures, and this is of particular importance, as telomeres have a significant impact on the occurrence of cancer, cell senescence, and apoptosis.

D_2_O reduces the number and stability of G-quadruplex structures, which provides a new demonstration to prove that D_2_O is not conducive to the survival, growth, and reproduction of organisms [37,38,39] but can lead to the occurrence of genetic mutations [40] and other deleterious physiological effects. In addition, D_2_O can also be used as a “regulatory switch” for G-quadruplexes, which could reduce the D_2_O content around the sequence when more G-quadruplexes are needed. Furthermore, the amount of D_2_O around the sequence would be decreased when fewer G-quadruplexes are needed, which would have potential applicability in research, including genetics, in vitro studies, and nucleotide medicine.

The above conclusion highlights the significance of studies on the interaction between D_2_O and ssDNA. Here are six conclusions that summarize the interactions between D_2_O and ssDNA. 1. D_2_O may have the following effects on DNA transcription and replications: (i) A small exchange of hydrogen and deuterium alters the bond energy and molecular activity of some bonds. (ii) The deuterium bond formed between the DNA molecule and D_2_O is stronger than the hydrogen bond, and the binding strength between DNA molecules, and consequently, the surface water, changes. (iii) D_2_O could alter the number of secondary structures such as G-quadruplexes that form part of the DNA, and these changes could affect DNA transcription, replication, and other processes. 2. The dsDNA forms numerous intramolecular hydrogen bonds through base pair complementation, but numerous groups can form intermolecular deuterium bonds with environmental D_2_O. Both RNA and ssDNA are single-stranded structures, and their interactions with D_2_O should be similar. Therefore, this study could enhance our understanding of the interactions between dsDNA, RNA, and H_2_O/D_2_O. 3. Oligonucleotide drugs are relatively stable, but it is also important to ensure stability during long-term storage and transportation. The interaction between D_2_O and ssDNA could change the activity of some groups. For some ssDNAs that can form a specific secondary structure, the number of secondary structures can also be changed. This can affect the interaction of ssDNA with other substances in the environment, thereby ensuring its stability. 4. Nucleic acid aptamers can bind to ligands with high affinity and strong specificity, which makes the slight change in the aptamer have a remarkable influence on binding. The effect of D_2_O on nucleic acids is almost constant, where there is a small hydrogen–deuterium exchange and numerous intermolecular hydrogen bonds, and the number of secondary structures is affected, which can serve as a means to appropriately alter the aptamer. This would increase the power and specificity of nucleic acid aptamers and ligand recognition, improving the sensitivity and specificity of aptamer-based biosensing. 5. The number of active H atoms or the number and type of groups capable of forming H bonds in different compounds vary; thus, the strength of the interaction between the bonds and D_2_O is theoretically different. By determining the intensity of the SERS peak or the degree of shift change, the active H of the compound and the number or type of groups that can form H bonds can be compared to identify the compound and determine whether the structure of the compound has changed. 6. Biological macromolecules contain numerous active H groups capable of forming hydrogen bonds, which are D_2_O active sites. As D_2_O and these active sites basically exhibit the same activity, an understanding of the interaction between D_2_O and ssDNA could provide a reference when studying the interaction between D_2_O and other biological macromolecules.

## 3. Materials and Methods

### 3.1. Samples and Reagents

ssDNA was purchased from Sangon Biotech Co., Ltd. (Shanghai, China), and Table 1 shows the sequences. Analytically pure silver nitrate (AgNO_3_), sodium citrate (Na_3_C_6_H_5_O_7_·2H_2_O), and nitric acid (HNO_3_) were purchased from Sinopharm Chemical Reagent Co., Ltd. (Shanghai, China). Analytically pure potassium iodide (KI) and MgSO_4_ and all other experimental reagents were purchased from Shanghai Titan Technology Co., Ltd. (Shanghai, China). Deionized water was used for all the experiments.

### 3.2. Instruments and Equipment

A Renishaw inVia confocal Raman spectrometer (Renishaw plc, London, UK), high-speed centrifuge (TG16-WS, Shanghai Lu Xiangyi Centrifuge Instrument Co., Ltd., Shanghai, China), multifunctional vortex mixer (Vortex-Genie2, Scientific Industries, Inc., New York, NY, USA), laboratory pure water system (Smart-D UV, Shanghai Hetai Instrument Co., Ltd., Shanghai, China), collector-type constant-temperature heating magnetic stirrer (ZNCL-TS, Shanghai Yike Instrument Co., Ltd., Shanghai, China), and an electronic balance are some of the main equipment used.

### 3.3. Sample Processing

In this experiment, the classic method of Lee [41] was used to prepare a nano-silver collagen solution, which was subsequently stored at 26 °C for 7 days before use. Appropriate volumes of water and D_2_O were added to the powdered ssDNA to prepare sample solutions with D_2_O proportions of 0%, 18.75%, 37.5%, 56.25%, 75%, and 100%. The ssDNA concentration was 25 μM. The prepared sample solutions were placed in a 90 °C water bath for 10 min, cooled to room temperature, and then placed at a temperature of 4 °C for 48 h. Then, 10 mL of silver collagen solution was centrifuged (7000 rpm, 10 min), and 100 μL of the supernatant was added to the same volume of KI (1 mM) solution, thoroughly mixed, and then incubated at room temperature for 30 min to clean the surface of the silver nanoparticles for later use. Furthermore, 5 μL of a concentrated gel was placed in an EP tube and mixed thoroughly with 5μL of the sample solution, followed by the addition of 0.8 μL of MgSO_4_ (20 mM) solution, and the mixture was allowed to stand before it was detected.

### 3.4. Raman Detection and Microscopy

A Renishaw inVia Raman confocal microscope coupled to a Leica DMi8/SP8 laser scanning confocal microscope system with a 532 nm diode laser as the excitation source (power of 25 mW) was directed at the sample, and an 1800 L/mm grating was used for the measurements. The laser was focused on the sample using an xL50 objective.

### 3.5. Data Processing

The collected raw data were normalized with the 1091 cm^−1^ peak (attributable to PO_2_^−^ stretching vibration) as the standard. The spectra were not smoothed before they were graphed using the Origin software. Linear data were obtained by normalization and background subtraction.

## 4. Conclusions

ssDNA interacts with D_2_O in the environment, thereby affecting its structure and function. D_2_O mainly interacts with ssDNA through the A and G bases, and the higher their number, the greater the influence of D_2_O on the sequence structure. In the absence of certain specific secondary structures, the positions of the A and G bases in the sequence had almost no effect on the D_2_O–ssDNA interaction. Furthermore, D_2_O showed an inhibitory effect on the formation of the G-quadruplex structure based on the sequence. The interaction between the two can be leveraged to develop various new functional strategies and be used for the determination of secondary structures. ssDNA is not the only biomacromolecule of interest, as the interaction of RNAs (and proteins) with water also plays a significant role in determining their stability. In the future, SERS could be used to further explore the interactions of RNA, protein, and water.

## Figures and Tables

**Table 1:** Single-stranded DNA (ssDNA) sequences and the sequences they represent.

Name	Sequence (5′-3′)	Name	Sequence (5′-3′)
SJ16	GTGAGCTGGCGGCAAC	G12ATC	GAGCGATGGGAGCGGAGTGG
SJ20	CAGGTCCAGGCTGCAGGTAG	A4GTC	ATCCGATCCGATCCGATCCG
A20	AAAAAAAAAAAAAAAAAAAA	A8GTC-1	ATCAGATCAGATCAGATCAG
G14	GGGGGGGGGGGGGG	A8GTC-2	ATACGATACGATACGATACG
C20	CCCCCCCCCCCCCCCCCCCC	A8GTC-3	TACAGTACAGTACAGTACAG
T20	TTTTTTTTTTTTTTTTTTTT	A12GTC	ATAAGAACAGAACAGATAAG
T10C10	TTTTTTTTTTCCCCCCCCCC	(G_3_T)_4_	GGGTGGGTGGGTGGGT
TC20	TCTCTCTCTCTCTCTCTCTC	TG_7_T	TGGGGGGGT
G4ATC	ATCCGATCCGATCCGATCCG	Tel21	GGGTTAGGGTTAGGGTTAGGG
G8ATC-1	ATCGGATCGGATCGGATCGG	93del	GGGGTGGGAGGAGGGT
G8ATC-2	ATGCGATGCGATGCGATGCG	GO18	AACCTTTGGTCGGGCAAGGTAGGTT
G8ATC-3	AGTCGAGTCGAGTCGAGTCG	GO18T	TTGGTCGGGCAAGGTAGGTT

## Data Availability

The data presented in this study are available on request from the corresponding author.

## Sample Availability

Samples of the compounds are available from the authors.
